# Pruritus and protein-bound uremic toxins in patients undergoing hemodialysis: a cross-sectional study

**DOI:** 10.1093/ckj/sfae007

**Published:** 2024-01-09

**Authors:** Suguru Yamamoto, Takahiro Tanaka, Kentaro Omori, Isei Ei, Kaori Kikuchi, Ayano Konagai, Shin Goto, Nobutaka Kitamura, Ichiei Narita

**Affiliations:** Division of Clinical Nephrology and Rheumatology, Niigata University Graduate School of Medical and Dental Sciences, Niigata, Japan; Clinical and Translational Research Center, Niigata University Medical and Dental Hospital, Niigata, Japan; Omori Clinic, Niigata, Japan; Santo-Second Clinic, Niigata, Japan; Research and Development Division, Kureha Corporation, Tokyo, Japan; Research and Development Division, Kureha Corporation, Tokyo, Japan; Division of Clinical Nephrology and Rheumatology, Niigata University Graduate School of Medical and Dental Sciences, Niigata, Japan; Clinical and Translational Research Center, Niigata University Medical and Dental Hospital, Niigata, Japan; Division of Clinical Nephrology and Rheumatology, Niigata University Graduate School of Medical and Dental Sciences, Niigata, Japan

**Keywords:** 5D-itch scale, hemodialysis, indoxyl sulfate, pruritus, uremic toxins

## Abstract

**Background:**

Patients undergoing hemodialysis frequently experience pruritus; its severity is associated with poor quality of life and mortality. Recent progress in hemodialysis treatment has improved the removal of small- and middle-molecular-weight molecules; however, the removal of protein-bound uremic toxins (PBUTs) remains difficult. It is possible that pruritus is associated with serum PBUTs in patients undergoing hemodialysis.

**Methods:**

We conducted a multicenter cross-sectional study in patients undergoing hemodialysis (*n* = 135). The severity of pruritus was assessed using the 5D-itch scale and medication use. Serum PBUTs, including indoxyl sulfate, p-cresyl sulfate, indole acetic acid, phenyl sulfate, and hippuric acid, were measured using mass spectrometry; the PBUT score was calculated from these toxins using principal component analysis. Univariate and multiple regression analyses were performed to examine independent predictors of pruritus.

**Results:**

Pruritus was reported by 62.2%, 21.5%, and 13.3%, 1.5% and 0.7% as 5 (not at all), 6–10, 11–15, 16–20, and 21–25 points, respectively. The PBUT score was higher in patients undergoing dialysis having pruritus than those without pruritus (0.201 [–0.021 to 0.424] vs –0.120 [–0.326 to 0.087]; *P* = 0.046). The PBUT score was shown to have an association with the presence of pruritus (coefficient 0.498$\ \pm \ $0.225, odds ratio: 1.65 [1.06–2.56]; *P* = 0.027).

**Conclusion:**

Uremic pruritus was frequently found and associated with the PBUT score in patients undergoing hemodialysis. Further studies are required to clarify the impact of PBUTs on uremic pruritus and to explore therapeutic strategies in patients undergoing hemodialysis.

KEY LEARNING POINTS **What was known:**

- Patients undergoing dialysis frequently develop pruritus, even with the progress of dialysis treatment and medication.
- Protein-bound uremic toxins (PBUTs) are difficult to remove using conventional dialysis.
- Thus, exposure to various PBUTs may be associated with uremic pruritus.

**This study adds:**

- Pruritus was reported by 38% of patients undergoing hemodialysis.
- We developed a PBUT score based on the five types of PBUT, and it was associated with the presence of pruritus.

**Potential impact:**

- Comprehensively evaluating PBUTs and increasing their removal during dialysis treatment may effectively improve pruritus in patients on dialysis.

## INTRODUCTION

Patients with chronic kidney disease (CKD), especially those undergoing dialysis, have poor quality of life (QOL) due to various systemic disorders compared with the general population [1, 2]. Among them, pruritus is one of the most frequent complications [3, 4]. Severity is associated with poor QOL [5–7] but also worse mortality in patients with CKD [6, 8, 9]. A previous study suggested that the severity of CKD-related pruritus is associated with serum calcium, phosphate, and β_2_-microglobulin (β2m) in patients undergoing hemodialysis (HD) [9]. Recent progress in dialysis treatment using high-flux dialyzers and online hemodiafiltration (HDF) has improved the removal of small- and medium-molecular-weight molecules. Medications including phosphate binders, active vitamin D, and calcimimetics facilitate the management of mineral and bone disorders. However, even with progress in dialysis treatment and medication, uremic pruritus remains a critical complication, probably due to other CKD-specific factors.

The accumulation of protein-bound uremic toxins (PBUTs) is a major CKD-specific factor that induces various systemic disorders [10–14]. Several molecules, such as indoxyl sulfate (IS) and p-cresyl sulfate (PCS), show high protein-binding properties and are difficult to remove using conventional dialysis [15]. Because various PBUTs continue to accumulate in patients with end-stage kidney disease (ESKD), multiple exposures to PBUTs may affect the pathology of pruritus.

This study aimed to evaluate the severity and characteristics of pruritus in patients undergoing maintenance HD. We also examined the association between pruritus and PBUTs.

## MATERIALS AND METHODS

### Study design

Adult patients undergoing maintenance HD who visited three outpatient clinics were eligible for inclusion in this cross-sectional study. Patients aged <20 or >100 years and those who could not answer the pruritus and QOL questionnaires by themselves were excluded. Between August 2017 and April 2018, patients were invited to participate in the study and provided informed written consent independently. The severity of pruritus was assessed using the 5D-itch scale, visual analog scale (VAS), body part affected, and use of medication for pruritus. Serum samples before the dialysis session were collected on the same day as the pruritus survey. This study adhered to the Declaration of Helsinki and was approved by the Central Ethics Committee of Niigata University (No. 2016–0005). This study was registered at the University Hospital Medical Information Network Center (UMIN000028464). All the participants provided written informed consent.

### Patient characteristics

Demographic data were obtained from individual patient files: age, sex, body mass index (BMI), systolic and diastolic blood pressure, dialysis vintage, the primary cause of CKD, dialysis modality (HD or HDF), HD treatment adequacy (Kt/V_urea_), blood hemoglobin, serum albumin, serum calcium, serum phosphate, serum c-reactive protein, β2m, and intact parathyroid hormone (PTH) were included. It was determined that residual kidney function was present when the urine volume exceeded 200 mL/day. QOL was assessed using the Kidney Disease Quality of Life Short Form (KDQOL-SF, Version 1.3) [16, 17]. Kidney disease and physical, mental, and social component summaries were recorded.

### Assessment of pruritus

The severity of pruritus was measured using the 5D-itch scale, VAS, and the frequency of medication use at baseline. The 5D-itch scale has previously been translated into Japanese and validated in patients with CKD [18, 19]. The scoring system comprised five domains: degree, duration, direction, disability, and distribution. The distribution of body parts with itching was as follows: head/scalp, face, chest, abdomen, back, buttocks, thighs, lower legs, top of feet/toes, soles, palms, top of hands/fingers, forearms, upper arms, points of contact with clothing, and groins. The scores of each of the five domains were summed to obtain a total score ranging from 5 (no pruritus) to 25 (most severe pruritus). VAS was used to validate the accuracy of the 5D itch scale (VAS: 0, no pruritus; 10, maximal pruritus) [20]. Medications for pruritus included external heparinoids, antihistamines (external or oral), steroids (external or oral), and nalfurafine. The presence of pruritus was considered on a 5D-itch scale of more than 6 points and/or the use of medications for pruritus (‘with pruritus’ group), and patients without pruritus were considered to have a 5D-itch scale score of 5 points without medication use (‘without pruritus’ group).

### Measurements of PBUTs

Serum specimens collected from patients and the reaction solutions were immediately frozen at –30°C and thawed just before the measurement of PBUTs. The respective levels of the total forms of the five PBUTs, IS, PCS, indole acetic acid (IAA), phenyl sulfate (PhS), and hippuric acid (HA), were measured using mass spectrometry, as described previously [15].

### Statistical analysis

Our sample size to detect Spearman's rank correlation coefficient (r = 0.3) with 90% power and a two-sided α of 0.05 was calculated as 135 patients. Results are expressed as mean (standard deviation) or median (interquartile range). Differences between the with and without pruritus groups were calculated using the unpaired *t*-test or *U*-test. Our interest in this study was the relationship between PBUTs and pruritus. Because those PBUTs may be associated with pruritus multiply, we calculated the ‘PBUT score’ from five kinds of PBUTs (IS, PCS, PhS, IAA, and HA) with principal component analysis. The principal component analysis is a multivariate analysis that shrinks multiple variables to fewer factors. Two sample statistical tests for each PBUT and a test for the principal component score using all PBUTs were performed to examine the independent predictors of the presence of pruritus. Uni- and multivariate logistic regression analyses were performed using the presence or absence of pruritus as the dependent variable. PBUT score, age (binary variable), sex, dialysis vintage (quartile group), dialysis modality (HD or HDF), and kidney disease component summary were the independent variables. We considered 2-sided *P* < 0.05 as statistically significant.

## RESULTS

### Study samples

This analysis included 135 patients on HD who responded to the 5D-itch scale and VAS scores. Participants’ characteristics are presented in Table 1. Male participants accounted for 75% of the total, and the mean age was 64.9 ± 12.1 years. Primary causes of CKD were diabetes (33%), chronic glomerulonephritis (55%), and nephrosclerosis (6%; Table 1). The median duration of dialysis treatment was 89 (33–194) months, and single-pool Kt/V_urea_ was 1.5 (1.3–1.7) (Table 1).

**Table 1:** Patient characteristics of pruritus in hemodialysis patients

	All	Without pruritus	With pruritus	*P* value
No. of patients	135	84 (62%)	51 (38%)	
Severity of pruritus				
5D-itch scale	5 [5–8]	5 [5–5]	8 [8–11]	0.001
VAS	0.5 [0.0–3.9]	0.0 [0.0–0.5]	3.7 [1.4–7.0]	0.001
Case-mix				
Age, years	64.9 ± 12.1	65.6 ± 11.2	63.8 ± 13.5	0.391
Male sex	101 (75%)	60 (71%)	41 (80%)	0.245
Cause of ESKD, DM/CGN/others				
Diabetic nephropathy	45 (33%)	21 (25%)	24 (47%)	0.067
Chronic glomerulonephritis	74 (55%)	51 (61%)	23 (45%)	
Nephrosclerosis	8 (6%)	6 (7%)	2 (4%)	
Others	8 (6%)	6 (7%)	2 (4%)	
BMI, kg/m^2^	21.3 [19.7–24.2]	21.0 [1.95–23.9]	22.2 [19.8–24.4]	0.252
Systolic blood pressure, mmHg	145 ± 22	145 ± 24	146 ± 19	0.842
Diastolic blood pressure, mmHg	74 ± 14	74 ± 15	74 ± 13	0.992
Residual kidney function	16 (11.9%)	10 (11.9%)	6 (11.8%)	0.981
Dialysis treatment				
Vintage, months	89 [33–194]	89 [46–200]	90 [27–167]	0.339
Single pool Kt/V	1.5 [1.3–1.7]	1.5 [1.3–1.7]	1.4 [1.2–1.7]	0.509
Hemodiafiltration	37 (27%)	16 (19%)	21 (41%)	0.005
Laboratory values				
Hemoglobin, g/dL	10.4 ± 1.1	10.4 ± 1.2	10.4 ± 1.1	0.967
Albumin, g/dL	3.6 ± 0.4	3.6 ± 0.4	3.6 ± 0.4	0.869
C-Reactive protein, mg/dL	0.1 ± 0.3	0.1 ± 0.3	0.1 ± 0.4	0.482
Calcium, mg/dL	8.8 ± 0.8	8.8 ± 0.8	8.8 ± 0.7	0.691
Phosphate, mg/dL	5.3 [4.5–6.1]	5.2 [4.2–6.2]	5.5 [4.7–6.1]	0.468
Parathyroid hormone, pg/mL	180 [93–221]	162 [92–221]	184 [111–231]	0.536
β_2_-microglobulin, mg/L	28.8 ± 6.3	28.8 ± 6.3	28.7 ± 6.3	0.864
Indoxyl sulfate, mg/dL	2.73 [2.03–3.90]	2.58 [1.96–3.70]	2.98 [2.32–4.06]	0.084
Phenyl sulfate, mg/dL	0.80 [0.44–1.17]	0.80 [0.43–1.30]	0.79 [0.50–1.11]	0.967
p-Cresyl sulfate, mg/dL	1.80 [0.70–3.21]	1.69 [0.63–2.93]	2.17 [1.00–3.50]	0.181
Hippuric acid, mg/dL	4.18 [1.87–6.91]	3.80 [1.50–6.78]	4.51 [2.13–7.12]	0.395
Indole acetic acid, mg/dL	0.09 [0.06–0.15]	0.10 [0.06–0.15]	0.09 [0.06–0.14]	0.618
KD-QOL				
Kidney disease component summary	65.5 [63.8–67.2]	65.1 [62.9–67.3]	66.2 [63.3–69.0]	0.906
Physical component summary	32.2 [29.5–34.9]	31.6 [28.0–35.1]	33.3 [29.2–37.4]	0.536
Mental component summary	53.8 [51.8–55.8]	54.3 [51.9–56.6]	52.9 [49.2–56.6]	0.509
Social component summary	40.8 [37.1–44.5]	40.8 [36.0–45.6]	40.9 [34.6–47.0]	0.992

### Descriptive data of pruritus

**Figure 1:**
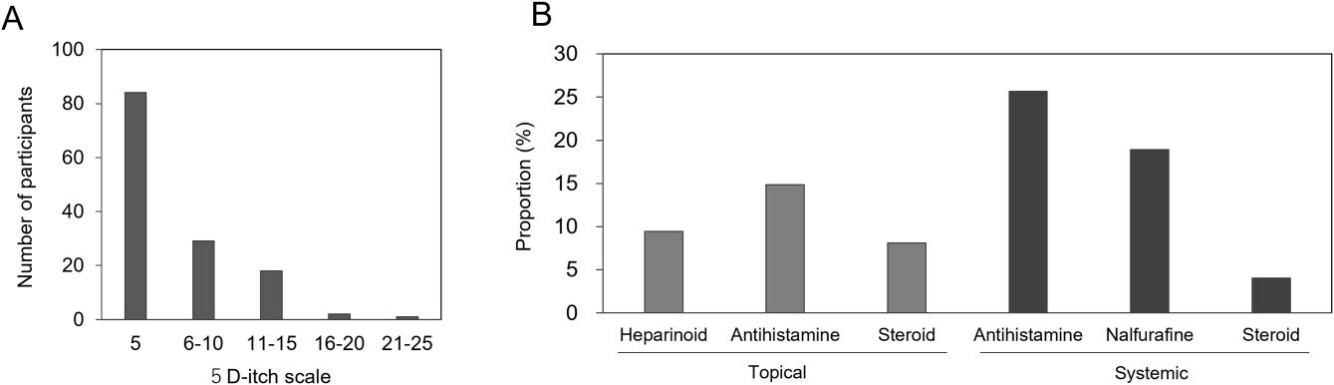
Prevalence of uremic pruritus in patients undergoing hemodialysis. **A** Severity of pruritus is assessed by 5D-itch scale in hemodialysis patients. Data are presented with five categories, including 5 (not at all), 6–10, 11–15, 16–20, and 21–25 points. **B** Proportion of medication use for uremic pruritus. Percentages of medications used, including topical antihistamines, topical corticosteroids, topical heparinoids, oral antihistamines, nalfurafine, and oral corticosteroids, are shown for patients with pruritus.

Pruritus, measured using the 5D-itch scale, was reported by 62.2%, 21.5%, 13.3%, 1.5%, and 0.7% as 5 (not at all), 6–10, 11–15, 16–20, and 21–25 points, respectively (Fig. 1A). The median 5D-itch scale and VAS scores were 5 [5–8] and 0.5 (0.0–3.9), respectively, and the 5D-itch scale was associated with VAS (ρ = 0.662, *P* < 0.001, Spearman's rank correlation coefficient). Body parts in patients who frequently had itching included the back (54.8%), lower leg (22.6%), thigh (19.0%), and upper arm (17.9%; Table 2). The patients with pruritus received medications including topical antihistamines (14.9%), topical corticosteroids (8.1%), topical heparinoids (9.5%), oral antihistamines (25.6%), oral nalfurafine (18.9%), and oral corticosteroids (4.1%; Fig. 1B). When the participants were divided into two groups, including the patients with a 5D-itch scale of more than 6 points and/or use of medications for pruritus (‘with pruritus’ group; *n* = 51; 38%) and the patients with a 5D-itch scale of 5 points without medication use (‘without pruritus’ group; *n* = 84; 62%), there was no difference in the patient characteristics, except for dialysis modality (Table 1). The ‘with pruritus’ group underwent HDF more frequently compared with the ‘without pruritus’ group.

**Table 2:** Spatial pattern of pruritus in hemodialysis patients

Distribution	*n* (%)
Head/scalp	10 (11.9)
Face	7 (8.3)
Chest	5 (6.0)
Abdomen	14 (16.7)
Back	46 (54.8)
Buttocks	4 (4.8)
Thighs	16 (19.0)
Lower legs	19 (22.6)
Tops of feet/toes	4 (4.8)
Soles	3 (3.6)
Palms	3 (3.6)
Tops of hands/fingers	1 (1.2)
Forearms	13 (15.5)
Upper arms	15 (17.9)
Points of contact with clothing	3 (3.6)
Groin	1 (1.2)

### Pruritus and PBUTs

Serum levels of IS, PCS, PhS, IAA, and HA at the predialysis session were 2.73 (2.03–3.90), 1.80 (0.70–3.21), 0.80 (0.44–1.17), 0.09 (0.06–0.15), and 4.18 (1.87–6.91) mg/dL, respectively, which were similar to those in previous reports [15, 21, 22]. There were no significant associations of each serum PBUT level between patients with and without pruritus (Table 1).

**Figure 2:**
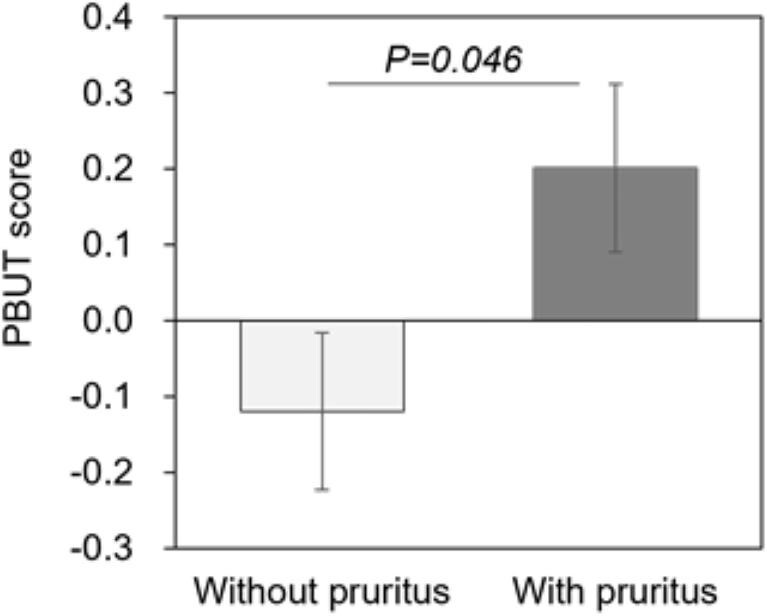
The protein-bound uremic toxin (PBUT) score and pruritus. The PBUT score was developed with principal component analysis and compared between hemodialysis patients with or without pruritus. Data are shown as the mean $ \pm $ standard deviation (error bars).

As uremic toxins may induce pruritus through multiple pathways, we calculated the PBUT score from IS, PCS, PhS, IAA, and HA using principal component analysis (Table S1 and Fig. S1, see online supplementary material). The factor scores for IS, PCS, IAA, PhS, and HA were 0.889, 0.346, –0.173, 0.285, and 0.509, respectively. The patients without residual kidney function had higher PBUT scores than those without residual kidney function (Table 2, see online supplementary material). The PBUT score was higher in patients with pruritus than in those without (0.201 [–0.021 to 0.424] vs –0.120 [–0.326 to 0.087]; *P* = 0.046; Fig. 2). Multivariate analysis showed the PBUTs score to have a significant association with pruritus adjusted for age (above or below median), sex, dialysis vintage (quartile group), dialysis modalities (HD or HDF), and the kidney disease component summary (coefficient 0.498$\ \pm \ $0.225; odds ratio 1.65 [1.06–2.56]; *P* = 0.027; Table 3).

**Table 3:** Association of pruritus with clinical factors

	Univariate					Multivariate				
Independent variables	Coefficient	SE	Lower of 95% CI of coefficient	Upper of 95% CI of coefficient	*P* value	Coefficient	SE	Lower of 95% CI of coefficient	Upper of 95% CI of coefficient	*P* value
PBUT score	0.386	0.19	0.014	0.758	0.042	0.498	0.2251	0.057	0.939	0.027
Male sex	−0.381	0.2026	−0.778	0.016	0.060	0.477	0.437	−0.38	1.333	0.275
Age >66 years old	−0.734	0.2483	−1.221	−0.247	0.003	−0.484	0.3813	−1.231	0.263	0.204
Dialysis modality (HDF)	−0.230	0.1347	−0.495	0.034	0.087	1.622	0.4641	0.713	2.532	<0.001
Dialysis vintage	−0.195	0.0667	−0.326	−0.064	0.004	−0.485	0.2034	−0.884	−0.087	0.017
Kidney disease component summary	−0.008	0.0027	−0.013	−0.002	0.004	−0.023	0.0103	−0.043	−0.003	0.024

HD, hemodialysis; HDF, hemodiafiltration; OR, odds ratio; PBUT, protein-bound uremic toxin.

## DISCUSSION

This multicenter cross-sectional study reported the characteristics of pruritus and its association with PBUTs in patients undergoing maintenance HD. Even though dialysis therapy and treatment for pruritus have progressed, 38% of patients had pruritus, which was associated with the PBUT score.

It is well known that uremic pruritus is a common complication in dialysis patients [3, 4]. A previous Dialysis Outcomes and Practice Patterns Study (DOPPS) from 2009 to 2018 reported that 40–50% of patients with HD had moderate-to-extreme itching when choosing between four degrees [5, 23]. In this study, we evaluated the severity of pruritus with the 5D-itch scale (a multidimensional measurement scale for pruritus) and found that 38% of patients had somewhat-to-severe pruritus, mainly in the back and lower leg (Fig. 1 and Table 2). The recent advancements in dialysis and pruritus treatments may influence the severity of pruritus in current maintenance HD patients. However, pruritus remains a common complication associated with ESKD.

Previous clinical studies have suggested several factors associated with uremic pruritus [9, 24]. In this study, despite advancements in dialysis therapy, pruritus was reported in 38% of cases. Contrary to factors previously reported in the literature (Kt/V, β2m, calcium, phosphorus, and PTH), our study did not identify an association between these factors and pruritus in HD patients (Table 1). The improvement in the management (standardization) of water-soluble small and middle-sized molecules, possibly due to improved purification of dialysate and dialyzer performance, as well as the effective control of mineral and bone disorders through the use of phosphate binders, active vitamin D and calcimimetics, may have contributed to this discrepancy. Therefore, we considered several unmeasured and unresolved factors related to pruritus. We hypothesized that the accumulation of PBUT is a possible cause of pruritus in dialysis patients. PBUTs are difficult to remove using conventional dialysis treatment [15] and are associated with several CKD-related systemic disorders, such as all-cause mortality [10–12], infectious events [10], and cognitive disorders [25]. In non-dialysis CKD patients, serum PCS levels are associated with pruritus, as measured by the VAS and 5D-itch scale [26].

Our results showed that the PBUT score developed from the five types of PBUT using principal component analysis was associated with pruritus (Table 3). Multiple exposures to various PBUTs may exacerbate pruritus in dialysis patients, and the PBUT score may indicate the association of PBUTs on skin damage in these patients. There are several factors associated with serum PBUT levels in HD patients. Previous reports showed the association of serum IS and PCS levels with residual kidney function [27]. Our data also showed the association between the PBUT score and residual kidney function (Table S2, see online supplementary material). In addition, the content of the diet, nutritional status, and dysbiosis induced with CKD may also affect the values of PBUT [28].

In this study, HDF was associated with pruritus in the patients with maintenance dialysis treatment HDF is often chosen for patients with severe pruritus because it removes medium-sized molecules that are probably associated with pruritus. However, our data showed that undergoing HDF was associated with pruritus (Tables 1 and 3). One possible reason for this is that HDF is chosen for HD patients with pruritus, while the effect is not sufficient. Previous studies suggest that HDF does not show an improvement in pruritus compared to HD using a high-flux dialyzer, as in previous reports [29, 30]. While it may not serve as a contributing factor to pruritus, it is imperative to acknowledge that causation cannot be definitively established in the context of this cross-sectional study. Further exploration of this phenomenon is warranted, necessitating observational or intervention studies in the future. We uniformly recruited cases of HD patients from three dialysis facilities to assess pruritus and QOL. As a result, 75% of the patients were male, and 55% had a primary cause of ESKD of chronic glomerulonephritis, indicating a skewed patient background. Because there were no differences in patient backgrounds between the groups with and without pruritus, we believe that the association between PBUTs and pruritus was appropriately evaluated. While the study adequately established the number of participants based on sample size calculations, it is important to note that the investigation involved a small number of cases. Therefore, we acknowledge the need for future studies with a larger number of cases.

This study had several limitations. This cross-sectional study did not clarify the causal relationship between pruritus and clinical factors. The sample size may not have been sufficient because we calculated it according to a previous study on PCS and pruritus in patients with CKD not on dialysis [26]. We suggested the importance of the PBUT score when considering the total toxicity of PBUTs in CKD-related systemic disorders. However, we did not conduct a validation in this study. We regarded steroids as medications for pruritus; however, it is possible that they are used for pruritus and other diseases. Despite these limitations, this is the first report to suggest the importance of total PBUTs with pruritus in patients undergoing dialysis. Large-scale international studies are required to confirm these findings.

In conclusion, uremic pruritus was frequently observed in patients undergoing HD and was associated with the PBUT calculated using several serum PBUTs. Further studies are required to clarify the impact of PBUTs on uremic pruritus and to explore therapeutic strategies in patients undergoing HD.

## Supplementary Material

sfae007_Supplemental_File

## Data Availability

Data will be available immediately after publication with no end date. Data will be shared upon reasonable request to the corresponding author. Restrictions apply to the availability of the data analysed in this study to preserve patient confidentiality. Proposals should be directed to yamamots@med.niigata-u.ac.jp to gain access.
