# Supplementary material for: Pruritus and protein-bound uremic toxins in patients undergoing hemodialysis: a cross-sectional study
Source: Clin Kidney J. 2024 Jan 9;17(1):sfae007. doi: 10.1093/ckj/sfae007 (PMC10818225; doi:10.1093/ckj/sfae007)
Supplement: sfae007_Supplemental_File [file sfae007_supplemental_file.docx]

Supplemental Table 1. Factor scores of PBUTs in principal component analysis

| Variables | Factor score |
| --- | --- |
| Indoxyl sulfate | 0.889 |
| Phenyl sulfate | 0.285 |
| p-Cresyl sulfate | 0.346 |
| Hippuric acid | 0.509 |
| Indole acetic acid | -0.173 |

Factor extraction method: Maximum likelihood method

Eigenvalue: 1.821, Proportion (%): 36.4

Supplemental Table 2. Association between PBUT score and residual kidney function

| Residual kidney function | N | Mean PBUT score | SD | P value |
| --- | --- | --- | --- | --- |
| No | 118 | 0.076 | 0.844 | 0.008 |
| Yes | 16 | -0.558 | 1.129 |  |
| Total | 134 | 0.000 | 0.902 |  |


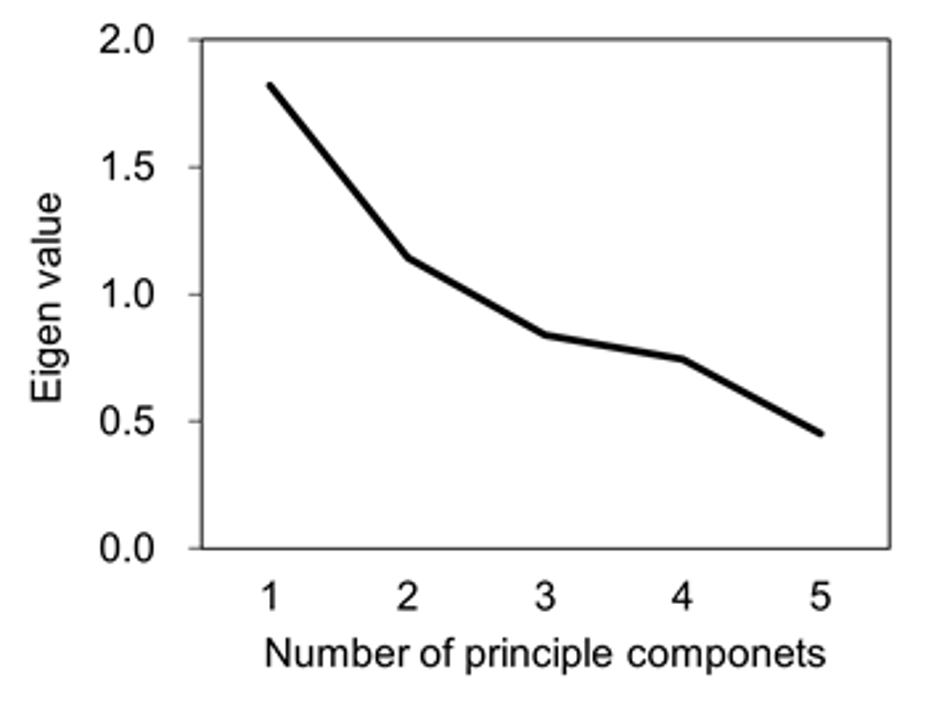
Supplemental Figure 1. Screw plot of principal component analysis in PBUTs

| Principle component | 1 | 2 | 3 | 4 | 5 |
| --- | --- | --- | --- | --- | --- |
| Eigen value | 1.821 | 1.142 | 0.84 | 0.744 | 0.453 |
| Proportion (%) | 36.4 | 22.8 | 16.8 | 14.9 | 9.1 |
| Cumulative proportion (%) | 36.4 | 59.3 | 76.0 | 90.9 | 100 |
